# Targeting dengue through mucosal vaccination: the potential of NS1 and bacterial spore-based delivery platforms

**DOI:** 10.3389/fimmu.2026.1809605

**Published:** 2026-07-14

**Authors:** Nurfatihah Zulkifli, Ok Sarah Shin, Sazaly AbuBakar

**Affiliations:** 1 Tropical Infectious Diseases Research and Education Centre (TIDREC), Universiti Malaya, Kuala Lumpur, Malaysia; 2 Institute for Advanced Studies, Universiti Malaya, Kuala Lumpur, Malaysia; 3 BK21 Graduate Program, Department of Biomedical Sciences, Korea University College of Medicine, Seoul, Republic of Korea; 4 Vaccine Innovation Center, Korea University College of Medicine, Seoul, Republic of Korea

**Keywords:** *Bacillus subtilis*, dengue, non-structural protein 1 (NS1), spores, vaccine

## Abstract

Severe dengue is characterized by marked vascular leakage, hemorrhagic manifestations, and organ impairment, largely driven by immunopathological mechanisms. Traditional vaccine approaches focusing on inducing neutralizing antibodies against DENV structural antigens have shown limitations and, under certain conditions, may also worsen infection through antibody-dependent enhancement (ADE) of infection. More recently, increasing evidence has highlighted the importance of T cell-mediated immunity and anti-non-structural protein 1 (NS1)-specific responses in suppressing DENV replication and reducing disease severity. DENV NS1, however, is known to be associated with severe dengue pathologies. In this context, immunization against NS1 offers a strategic advantage by eliciting immune responses that may contribute to early control of virus replication while simultaneously mitigating NS1-mediated vascular pathology. A vaccine delivery platform that would stimulate protective immune responses yet avoid the potential pathologic effects of NS1, hence, is needed. *Bacillus subtilis* spores antigen delivery platform stands out as a promising platform for its safety, stability, and inherent adjuvant properties. This review summarizes current advances in NS1-based dengue vaccine development and progress in the *Bacillus subtilis* spore-based immunization strategy against severe dengue.

## 1 Introduction

Dengue virus (DENV) infection remains one of the most significant arboviral threats worldwide, with an estimated 390 million infections annually (1). Despite decades of research on dengue vaccines and therapeutics, a universally accepted safe and effective vaccine and an effective antiviral therapeutic remain elusive. Current licensed vaccines such as Dengvaxia^®^ (CYD-TDV) and QDENGA^®^ (TAK-003) provide partial protection, with efficacy varying between serotypes, age groups, and pre-existing immunity. These limitations, coupled with a steadily increasing number of dengue cases worldwide, highlight the urgent need for alternative vaccine strategies that are both safe and capable of delivering broad, effective protection across diverse populations.

Among DENV proteins, the envelope (E) has been targeted as the primary antigen for vaccine development due to its involvement in virus attachment and fusion with host cell receptors. While E-specific antibodies are expected to prevent the virus from binding to its receptors, thereby inhibiting virus attachment, entry, and replication, this strategy has been complicated by the presence of highly cross-reactive epitopes on E that are non-neutralizing. Instead of preventing infection, these antibodies can bind to the virus and expose their Fc regions to Fcγ receptors (FcγR) on immune cell (2–4). The interaction with FcγR facilitates virus uptake and enhances virus replication, a phenomenon known as antibody-dependent enhancement (ADE) of infection (3, 5). ADE is particularly relevant during secondary infection with a heterologous DENV serotype, where pre-existing cross-reactive but non-neutralizing antibodies exacerbate viral replication and immunopathology, contributing to increased disease severity rather than conferring protection. Apart from that, the structural heterogeneity among DENV serotypes and conformational changes of E during virus maturation and host cell entry further complicate the design of a vaccine that is broadly neutralizing against all serotypes and variants.

Against this backdrop of challenges, DENV non-structural protein 1 (NS1) has gained attention as a strong vaccine candidate, demonstrating the ability to induce protective immunity without triggering the antibody-dependent enhancement (ADE) effect (6, 7). Given that NS1 is not a structural component of the virion, antibodies directed against NS1 do not bind virus particles and are therefore less likely to contribute to Fc receptor-driven ADE effects, which is commonly associated with anti-envelope antibodies. Several NS1-based vaccine strategies have been investigated, including nucleic acid delivery systems, recombinant fusion proteins, and peptide-based approaches. While NS1 is highly antigenic and could stimulate protective immunity, it is also able to cause severe pathologies. Hence, if NS1 is to be considered as a vaccine target, methods for its safe delivery are also needed. Among the vaccine delivery platforms being explored, those that would stimulate immunity through the mucosal route are emerging as novel alternatives, as they would not only target eliminating the virus but would also mitigate the potential of NS1-associated pathologies.

This review outlines key advances in dengue vaccine development, emphasizing the growing evidence of NS1 as a strong vaccine target and highlighting the potential of bacterial spores as effective mucosal vaccine delivery platforms. The bacterial spores-based vaccine platform, particularly that utilizing *Bacillus subtilis* spores, has emerged as a promising approach for mucosal antigen delivery, owing to its stability, safety, and intrinsic adjuvant properties. The review explores the potential of leveraging mucosal immunity to improve the effectiveness of dengue vaccines with particular emphasis on strategies that are both feasible and impactful for implementation in low- and middle-income countries (LMICs).

## 2 Replication and pathogenesis of dengue virus

Dengue fever is among the oldest recognized mosquito-borne viral diseases affecting humans. DENV, the causative agent of dengue fever, is a single-stranded, positive-sense RNA virus comprising three structural proteins: capsid (C), envelope (E), and membrane/pre-membrane (M/prM), and seven non-structural proteins: NS1, NS2A, NS2B, NS3, NS4A, NS4B, and NS5. The virus is transmitted to humans through the bites of infected female *Aedes aegypti* or *Aedes albopictus* mosquitoes. The infection typically begins when a mosquito’s saliva containing virus particles is injected through its proboscis into the dermis of the skin (8). At the entry sites, DENV primarily infects keratinocytes, fibroblasts, and dermal dendritic cells (DCs), including Langerhans cells (LCs). From the initial entry site, the virus replicates and subsequently disseminates to other organs. Infected LCs migrate from the epidermis to the lymph nodes, facilitating early dissemination and initiation of adaptive immunity (9). The initial virus replication is facilitated by mosquito salivary proteins. These proteins suppress innate antiviral responses in host immune cells by inhibiting inflammasome activation and cytokine release, thereby creating a favorable environment for the virus to replicate (10, 11). Mosquito salivary gland extract (SGE) was shown to increase virus particles in the dermis, enhance the infection of dendritic cells and macrophages, and promote their migration to lymph nodes (12). Mosquito salivary protein, such as the 34 kDa protein, suppresses interferon regulatory factors (IRFs), namely IRF-3 and IRF-7, while *Aedes aegypti* venom allergen-1 (AaVA-1) activates autophagy in host immune cells to facilitate viral replication (13, 14). This initial local virus amplification stage influences whether the virus will be effectively contained or become systemically disseminated.

**Figure 1:**
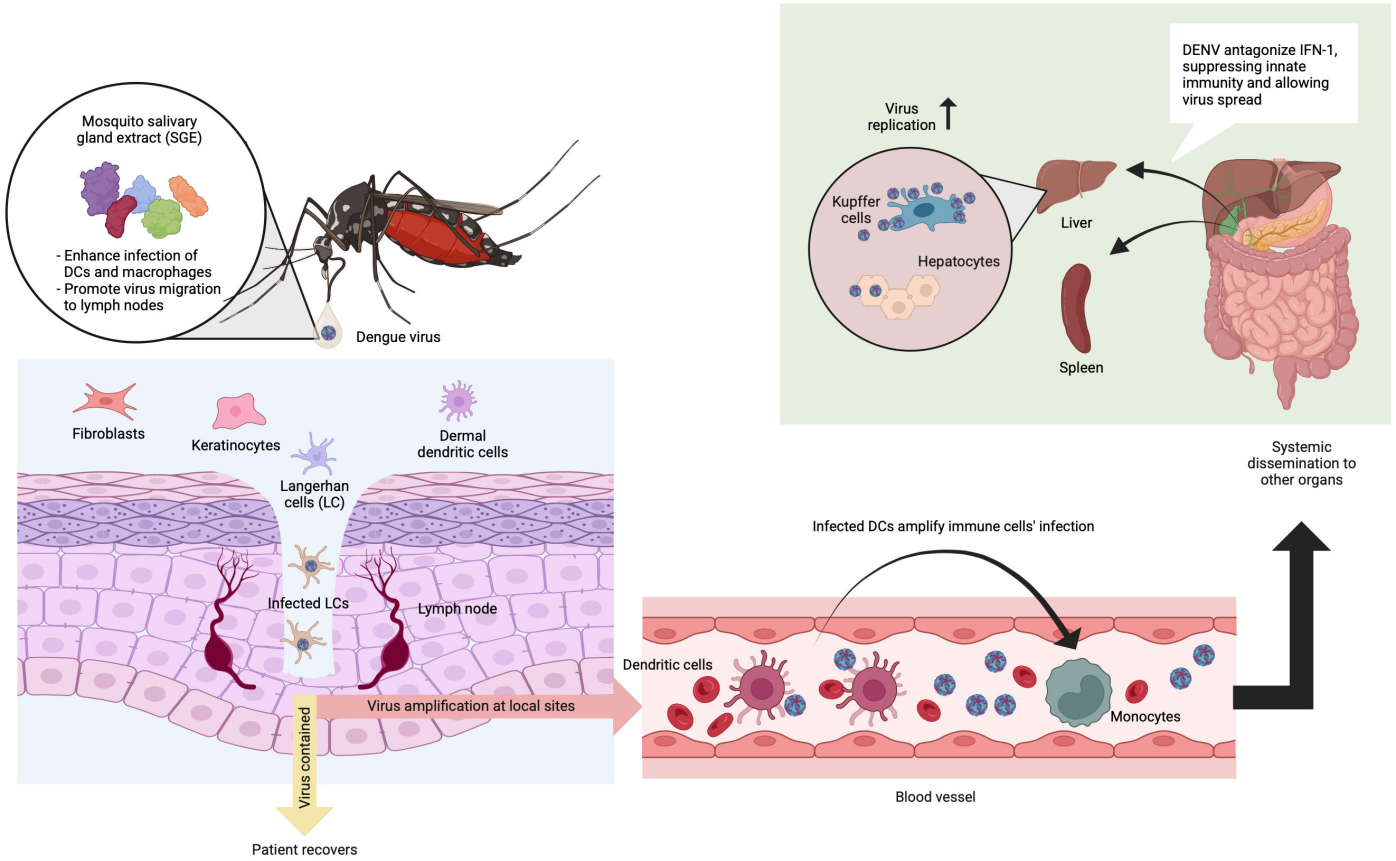
Initiation of DENV infection and its dissemination. Upon a mosquito bite, DENV and mosquito salivary gland extracts (SGEs) are introduced into the skin. SGE enhances DENV infection in skin cells, such as keratinocytes, fibroblasts, dermal dendritic cells, and Langerhans cells (LCs) by suppressing innate antiviral responses and facilitating early viral replication at local sites. If the local immune response successfully eliminates viral replication, the infection could be aborted or become asymptomatic. Conversely, if viral replication persists, infected LCs migrate to the lymph node, initiating early viral dissemination and triggering the initiation of adaptive immune responses. Once productive replication is established, DENV enters the bloodstream, resulting in systemic dissemination to other organs. This spread is facilitated by DENV non-structural proteins (NSs), which antagonize type I interferon (IFN-I) signaling to suppress innate immunity. Major DENV replication sites include the liver, where hepatocytes and Kupfer cells support robust virus replication, and the spleen, ultimately leading to the wide organ involvement characteristic of DENV infection. The image was created by Biorender.com.

Once productive local replication is established, DENV disseminates via the lymphatic system into the bloodstream, where it infects monocytes and myeloid DCs, facilitating viral spread to distant tissues (15). In particular, infected DCs facilitate the infection of monocytes and macrophages, thereby amplifying viremia and allowing virus spread to organs and tissues such as the liver, spleen, and vascular endothelium (15, 16). Viral suppression of innate immune defenses allows viruses to spread and cause subsequent infection in multiple tissues. DENV antagonizes type I interferon (IFN-I) signaling, particularly through the NS2A, NS2B, NS4A, NS4B, and NS5, disrupting innate immune defenses and allowing viruses to spread more efficiently (17–20). The liver is the major tissue target for DENV, where hepatocytes and Kupffer cells support productive virus replication (21). The spleen and lymph nodes are also important early sites of virus replication and lymphocyte activation (22, 23). Beyond these tissues, monocytes, macrophages, and DCs are major contributors to virus propagation and immune activation, whereas the infection of endothelial cells, despite being less productive, has significant consequences on vascular permeability (24, 25). Direct DENV infection has also been demonstrated in the bone marrow megakaryocytes, where it could impair platelet production and contribute to the development of thrombocytopenia (26–29). Moreover, bone marrow suppression, including reduced hematopoietic cells, has been observed, although its contribution to leukopenia remains incompletely defined (26, 27). This wide organ involvement in dengue underlies the systemic manifestations of DENV infection (**Figure 1**).

A hallmark of acute dengue is characterized by a high level of viremia, which plays a critical role in disease severity. Following an incubation period of approximately 4 to 7 days (ranging from 3 to 14 days), DENV RNA and infectious virions may become detectable in the blood, and this coincides with the onset of symptoms, including fever. Viremia typically peaks during the early febrile phase (days 1 to 4 of illness), which is followed by a gradual decline and defervescence, when fever subsides (days 4 to 6). Most patients would recover uneventfully within 7 to 10 days post-onset of fever. However, some patients may rapidly progress to severe manifestations, including plasma leakage, bleeding, and organ dysfunction. In a prospective cohort study conducted in Thailand, plasma leakage was observed in 47.7% of hospitalized patients with confirmed dengue infection, underscoring the importance of early recognition and appropriate clinical management (30, 31). Fluid reabsorption happens when patients start to recover through a mechanism that is still not well understood (32, 33). During acute infection, virus titers can reach over one million RNA copies per milliliter, correlating with disease severity and enhanced infectivity to mosquitoes upon a blood meal (34, 35). At such high titers, every mosquito that feeds on an acutely infected individual would become infected, potentially fueling subsequent extensive dengue outbreaks (36, 37). Severe dengue, however, is not only characterized by high viremia but also by immune-mediated events, including excessive cytokine responses and complement activation, leading to vascular dysfunction and intravascular leakage (34, 38). The mechanism of vascular leakage is thought to arise when DENV components and host-derived factors, such as cytokines, chemokines, growth factors, NS1, and antibodies, act on the vascular endothelium. Individuals with severe dengue have been shown to exhibit elevated levels of cytokines, including interleukin (IL)-4, IL-6, IL-8, IL-10, tumor necrosis factor-α (TNF-α), interferon-γ (IFN-γ), and monocyte chemoattractant protein-1 (MCP-1), along with reduced levels of IL-1β, IL-2, vascular endothelial growth factor (VEGF), and epidermal growth factor (EGF) (39, 40). Thus, while viremia reflects virus replication dynamics, the host’s exaggerated immune responses could also profoundly shape clinical outcomes.

## 3 Recent progress on dengue vaccine development

Despite vigorous national and regional dengue control efforts, the current strategies for dengue prevention, which mainly focus on vector control, behavioral changes, and community education, have proven inadequate. This is evident with the persistent increase in the number of reported dengue cases and dengue-related deaths globally (41). Most alarmingly, reports of dengue cases in regions where dengue was not previously recorded point to an expanding geographical presence of dengue (42). In addition, in regions where dengue has been endemic, a notable demographic shift has emerged in that young adults now represent the largest proportion of cases, replacing children as the primary affected group (43, 44). This highly active and mobile population represents a major source of DENV transmission during outbreak situations, considering that at least 60% of dengue infection is asymptomatic or mild despite being associated with a high level of viremia (45). Furthermore, high mortality among the elderly populations during dengue outbreaks has also been reported (46, 47). Hence, with the rapidly ageing population in Southeast Asia, where dengue has been endemic for more than four decades, the threat of dengue with high mortality is a serious public health concern. Thus, it is obvious that there is a clear and pressing need for strategies that can lower viremia during infection, reduce both disease severity and mortality, and curb DENV transmission.

Vaccination has been demonstrated as one of the most effective prevention strategies to reduce the burden of infection caused by many infectious agents. In relation to these, at least two dengue vaccines have been licensed: Dengvaxia^®^ (Sanofi Pasteur, France) and Qdenga^®^ (Takeda Pharmaceutical Company Limited, Japan). Research on the development of dengue vaccines, however, remains a continuous effort, primarily due to the daunting challenge of formulating a candidate vaccine capable of inducing consistent and protective immunity against each of the four DENV serotypes. Multiple dengue vaccine platforms have been developed, and several have already advanced into pre-clinical and clinical trials alongside the two currently licensed vaccines. These include live-attenuated vaccines, inactivated vaccines, DNA vaccines, mRNA vaccines, virus-like particle vaccines, recombinant subunit vaccines, and peptide-based vaccines (**Table 1**). Live-attenuated vaccines include CYD-TDV, TAK-003, and TV003/TV005. Although CYD-TDV and TAK-003 have been approved for use in a number of countries in the affected regions, their adoption has been lukewarm. CYD-TDV, a chimeric yellow fever-dengue vaccine, is only recommended for individuals aged 9 to 45 years with prior dengue exposure (67). This is due to its reported increased risk of developing severe dengue when used in seronegative individuals (49, 68). Despite inducing high neutralizing antibody titers, CYD-TDV demonstrated limited protective efficacy, particularly against DENV-2, and failed to prevent severe disease (50, 69, 70). More recently, Sanofi Pasteur, the manufacturer of the CYD-TDV vaccine, has announced plans to discontinue the manufacturing of the vaccine in the third quarter of 2026, citing low global demand (71). On the other hand, TAK-003, a live attenuated tetravalent vaccine based on a DENV-2 backbone, was recently introduced (72). The vaccine demonstrated a cumulative efficacy of ~ 61% against virologically confirmed dengue and ~ 84% against hospitalized cases (73). These data, however, suggest limited efficacy of the vaccine against DENV-3 in dengue-naïve recipients (73). The potential risk of enhanced DENV-associated diseases following DENV-3 or DENV-4 infection in baseline seronegative TAK-003 recipients also could not be excluded owing to the small sample size of the study (73). TV003/TV005, a vaccine developed by the U.S. National Institute of Allergy and Infectious Diseases (NIAID), was developed by introducing targeted deletions in the 3’ untranslated region (UTR) and mutations in non-structural proteins of the DENV (74). Early results from the clinical trials showed that TV003 induced strong and balanced immune responses against all four serotypes with a single immunization (75), while the modified version, TV005, provided stronger antibody responses following a single dose administration, especially against DENV-2, while maintaining its immunogenicity against the other DENV serotypes (76). The Butantan Institute in Brazil licensed TV003 and developed Butantan-DV, which has been evaluated in large-scale clinical trials (77). Phase 2 studies confirmed the safety of this vaccine, with most participants experiencing only mild rash while presenting high antibody responses against all four serotypes, particularly in seropositive volunteers (78). Preliminary results from an ongoing phase 3 clinical trial in Brazil have shown encouraging results, with overall vaccine efficacy at about 80%, with higher protection in seropositive individuals (78). The vaccine demonstrated strong efficacy against DENV-1 and moderate efficacy against DENV-2, while data on DENV-3 and DENV-4 are still being gathered due to limited circulation of these serotypes during the trial period (54). Meanwhile, Merck Sharp & Dohme (MSD, USA) is currently conducting the MOBILIZE-1 phase 3 study to assess the safety and efficacy of V181, a quadrivalent dengue vaccine candidate of TV003/TV005, in preventing dengue-associated disease in individuals aged 2 to 17 in the Asia-Pacific region (79).

**Table 1:** Current status of dengue vaccine candidates at various pre-clinical and clinical stages.

Platform	Candidate	Phase/stage	Developer	References
Live Attenuated	CYD-TDV	Licensed	Sanofi	(48–50)
	TAK-003	Licensed	Takeda	(51–53)
	TV003/TV005	III	^*^NIAID	(54, 55)
Inactivated	TDENV-PIV	II	^#^GSK/WRAIR	(56, 57)
DNA	D1ME100	I	^†^US NMRC	(58, 59)
mRNA	E80-mRNA, NS1-mRNA	I	^§^SPHCC, China	(60, 61)
VLPs	mD2VLP	Pre-clinical	^¶^NCKU, Taiwan	(62)
	AP205	Pre-clinical	Bern University, Switzerland	(63)
Recombinant Subunit	V180	I	^+^MSD	(64)
Peptide-based	PepGNP-Dengue	I	Emergex	(65)
	NP3	Pre-clinical	Sunway University, Malaysia	(66)

In contrast to live attenuated vaccines, inactivated dengue vaccines are produced by chemically or physically inactivating the virus and still preserving structural proteins (E, prM/M, C) for triggering immunogenicity. They are considered safer than the live-attenuated vaccines as they do not carry the risk of virus replication or reactivation, while also avoiding the issue of virus serotype interference that may arise in tetravalent live-attenuated formulations (80). The major drawback of this platform, however, is its limited immunogenicity, which arises from its inability to replicate in the host. As a result, the fixed, non-replicating antigen exposure to the immune system often requires the use of adjuvants in the vaccine formulation (81). On the other hand, TDENV-PIV, developed by GlaxoSmithKline (GSK) and the Walter Reed Army Institute of Research (WRAIR), includes all four DENV serotypes, which are inactivated with formalin to preserve antigenicity (82). Early clinical studies of the monovalent DENV-1 PIV vaccine demonstrated the safety and full seroconversion in all study participants with only mild reactions, such as injection-site pain (83). Phase I trials of the tetravalent TDENV-PIV with different adjuvants, including aluminum hydroxide, AS03B, and AS01E, demonstrated that the vaccine was well tolerated and produced balanced immune responses, with adjuvants AS03B and AS01E serving to augment robust antibody titers (84, 85). Ongoing phase 2 trials identified the optimal dosing schedule and reported some adverse events, including fatigue, headache, muscle aches, and fever, yet no severe complications were observed (56, 57).

Nucleic acid-based vaccines, including DNA and mRNA platforms, represent innovative approaches for dengue vaccine development. These strategies rely on host cells to synthesize virus antigens, which then stimulate the host immune responses and confer immunity (86). While DNA vaccines use plasmid vectors carrying antigen-encoding genes, mRNA vaccines directly deliver modified mRNA sequences packaged in lipid nanoparticles (LNPs) for expression in host cells. Both platforms offer advantages such as flexible antigen design, scalability through cell-free manufacturing, and relatively low production cost, but they differ in terms of safety considerations, immunogenicity, and delivery efficiency (87, 88). In particular, DNA vaccines are developed by inserting genes that encode virus antigens into a plasmid. For example, the DENV E stabilized by the prM is the main target because it mediates virus-host cell binding and elicits neutralizing antibodies. Upon administration through intramuscular injection, DNA plasmids could enter host cells directly or with the aid of delivery systems such as microneedles, lipid nanoparticles, or cationic polymers, which enhance cellular uptake (89–91). Once inside the host cells, the plasmids would direct the expression of viral antigens, which would then stimulate both innate and adaptive immunity. D1ME100 (TVDV/Vaxfectin^®^), a tetravalent DNA vaccine, was developed to include four plasmids encoding the prM and E of each DENV serotype. Pre-clinical studies in non-human primates and early human trials showed that it was safe and well-tolerated (92, 93). Its immunogenicity, however, was suboptimal, as even at high doses it generated only weak antibody responses, while low doses failed to induce adequate neutralizing antibodies (59, 92, 93). Dengue-specific IFN-γ-producing T cell responses, however, were detected in approximately 79% of high-dose recipients, but the limited breadth and uncertain protective capacity of these responses raised concerns regarding the quality of immunity induced by the vaccine. These limitations highlight the broader challenges associated with DNA vaccines, which include low immunogenicity in humans, inefficient cellular uptake without specialized delivery methods, and relatively weaker antibody responses, despite eliciting adequate T cell-mediated immunity. Compounding the complexity is the potential public hesitancy regarding the use of genetic vaccines driven in part by their theoretical risk of plasmid integration into the host genome (59, 94–97). Despite these drawbacks, DNA vaccines remain attractive due to their stability, cost-effectiveness, and scalability (58, 92). DNA vaccine efficacy may be improved through efficient promoters, advanced delivery systems, repeated dosing, and adjuvant co-immunization (80).

The mRNA vaccines are positioned as a significant advancement over DNA-based platforms, as they offer improved safety by eliminating concerns regarding potential genome integration and insertional mutagenesis (98, 99). Generally, mRNA vaccines consist of a 5’ cap, an open reading frame encoding target antigens, and a 3’ poly(A) tail, often delivered via LNPs (61). Several dengue mRNA vaccine candidates have been explored. An LNP-encapsulated vaccine encoding DENV-1 prM/E proteins was developed, inducing both humoral and cellular responses in AG129 murine model (60), whereas mRNA vaccines targeting DENV-2 prME, E80, and NS1 proteins demonstrated a robust production of DENV-specific neutralizing antibodies and T cell responses in mice (61). More recently, a multivalent vaccine candidate was engineered by combining NS1 and E-DIII sequences derived from different DENV serotypes (100). This multivalent mRNA vaccine demonstrated promising neutralizing activity against all four DENV serotypes with no evidence of significant ADE. These findings suggest that mRNA platforms may overcome some of the limitations of DNA vaccines. Rigorous clinical evaluation, however, is still needed to establish its long-term efficacy and safety.

Protein-based vaccines, including virus-like particles, recombinant subunit vaccines, and peptide-based vaccines, offer safer alternatives to live-attenuated and nucleic acid-based vaccines, as they rely on specific viral proteins or epitopes without involving virus replication (101, 102). Through antigen-specific targeting, protein-based vaccines mitigate concerns of virus replication, reversion to pathogenicity, and cross-serotype interference in multivalent designs. These platforms are highly adaptable, allowing for targeted immune responses against specific virus proteins, which can reduce the likelihood of ADE. This positions protein-based vaccines as strong candidates for dengue vaccine development, particularly when safety and targeted immune responses are priorities.

Recombinant subunit vaccines in general utilize virus genes expressed in prokaryotic or eukaryotic cells (102). Most dengue subunit vaccine candidates are based on truncated or modified E variants that aim to generate neutralizing antibodies to block DENV entry. As an example, the recombinant E candidate vaccine, V180, developed by Merck Sharp & Dohme (MSD USA), completed the phase 1 clinical trials (103). Pre-clinical studies in mice and non-human primates using V180 with ISCOMATRIX adjuvant demonstrated strong neutralizing antibody responses against all four DENV serotypes (104, 105). In human phase 1 trials, formulations with ISCOMATRIX were better tolerated and more immunogenic than those with aluminum-based adjuvants or no adjuvant (64). Despite these initial promising studies, there are still concerns with regard to protein misfolding due to the absence of post-translational modifications of the *Escherichia coli*-expressed antigen and concern over endotoxin contamination (106).In addition, insufficient data on T cell-mediated immunity from the initial studies emphasize the need for further optimization to ensure a robust and durable immunity.

Meanwhile, peptide-based vaccines consist of carefully selected epitopes from viral antigens, designed to elicit precise immune responses. Several epitope-based dengue peptide vaccines have been introduced. PepGNP-Dengue, a gold nanoparticle-based, multivalent synthetic peptide vaccine, has been developed and evaluated in phase I clinical trials (65). This vaccine candidate was designed to elicit CD8^+^ T cell-mediated immunity without inducing anti-DENV antibodies against structural proteins, particularly the E. Healthy volunteers receiving low doses of the peptides showed significant increases in CD8^+^ T cells and dengue-specific memory subsets, while high doses proved less effective, likely due to prolonged antigen retention at the injection site (65). Another study developed four multi-epitope peptides conjugated to nanoparticles, which elicited robust IgG and neutralizing antibody responses against all four DENV serotypes, while NP3 linked to the highly conserved B1 epitope derived from the E protein was able to elicit significant levels of IFN-γ (66). Based on results from these studies, protein or peptide-based dengue vaccines potentially offer safe and adaptable strategies that could target specific antigens while minimizing the risk of ADE. While pre-clinical studies show strong humoral and cellular responses, future strategies integrating structural design optimization, adjuvants, and multi-epitope targets could further enhance the breadth, durability, and cross-serotype protection of the next-generation protein-based dengue vaccines.

Virus-like particle (VLP) vaccines represent a distinct protein-based platform, consisting of structural proteins such as E and C that spontaneously assemble into VLPs, which resemble native virions in morphology but lack viral genetic material, thereby ensuring safety (107, 108). VLPs can be recognized by the immune system as a virus-like structure, triggering robust humoral and cellular immune responses. They can stimulate both neutralizing antibodies and T cell immunity, although adjuvants or multiple doses are often needed to enhance immunogenicity (108). A mature form of dengue VLP (mD2VLP) derived from DENV-2 was engineered to enhance the exposure of cryptic epitopes (62). Cryo-electron microscopy revealed that mD2VLP exhibited a T = 1 icosahedral symmetry with a diameter of approximately 31 nm, featuring a groove within the envelope protein dimers near 2-fold vertices (62). This structural configuration exposes previously hidden, quaternary structure-recognizing epitopes, increasing their accessibility to antibody recognition. Mice immunized with mD2VLP generated higher levels of cross-reactive neutralizing antibodies and were fully protected against all four DENV serotypes (62). In another pre-clinical study, researchers developed a combinatory vaccine using AP205 VLPs displaying EDIII from both Zika virus (ZIKV) and DENV (63). This approach aimed to provide broad protection against both viruses. The candidate vaccine induced a strong humoral immune response and neutralized all five targeted viruses after two doses, with no ADE observed (63). These findings highlight the potential of AP205 VLP-based combinatory vaccine as a promising approach for providing broad protection against flavivirus infections.

Overall, current dengue vaccine platforms remain constrained by incomplete serotype-balanced immunity, variability in host responses, and unresolved safety considerations associated with severe manifestations of dengue. These limitations are further compounded by the reliance on systemic immunity alone, with insufficient targeting of mucosal immune compartments that could contribute to broader immune priming. A comparative summary of the strengths and limitations of current dengue vaccine platforms is provided in **Table 2**. Although DENV is transmitted systemically by mosquito inoculation rather than mucosal surfaces, mucosal vaccination strategies could still enhance systemic immune readiness by promoting both mucosal IgA responses and systemic IgG and T-cell memory. Collectively, these gaps highlight the need for alternative vaccine strategies that can induce broader, safer, and more functionally diverse immune responses beyond conventional antibody correlates.

**Table 2:** Comparative assessment of major dengue vaccine platforms.

Platform	Key strengths	Major limitations	Safety concerns	Reasons for success/failure
Live Attenuated	Strong humoral and cellular immunity, mimic natural infection	Serotype imbalance, variable efficacy by serostatus, manufacturing complexity	Risk of severe disease in seronegative recipients, potential immune imbalance	Success linked to robust immune stimulation, shortcomings arise from unequal serotype replication and ADE-related concerns
Inactivated	Excellent safety profile, no risk of replication	Lower immunogenicity, require adjuvants and multiple doses	Generally mild adverse events	Safe but limited by inability to replicate and stimulate strong cellular immunity
DNA	Stable, scalable, cost-effective, induce cellular immunity	Weak antibody responses, inefficient uptake, multiple doses required	Theoretical concerns regarding genome integration, public acceptance issues	Failure largely attributed to low immunogenicity and inefficient antigen expression
mRNA	Rapid design and manufacturing, strong humoral and cellular responses in preclinical studies	Limited clinical data, storage and delivery challenges	Long-term safety and efficacy remain under evaluation	Success attributed to efficient antigen expression and flexibility
VLPs	Strong antigen presentation; induce humoral and cellular immunity, non-infectious	Manufacturing complexity, may require adjuvants	Favorable safety profile	Success linked to native-like antigen presentation
Recombinant Subunit	High safety, precise antigen targeting	Often weak cellular immunity, require adjuvants	Minimal safety concerns	Limited by suboptimal T-cell activation and potential protein-folding issues
Peptide-based	Highly specific, reduced ADE risk; flexible design	Limited immunogenicity, HLA restriction; require delivery systems	Generally favorable safety profile	Success depends on epitope selection and delivery platform

## 4 Dengue virus non-structural protein 1 as a vaccine target

**Figure 2:**
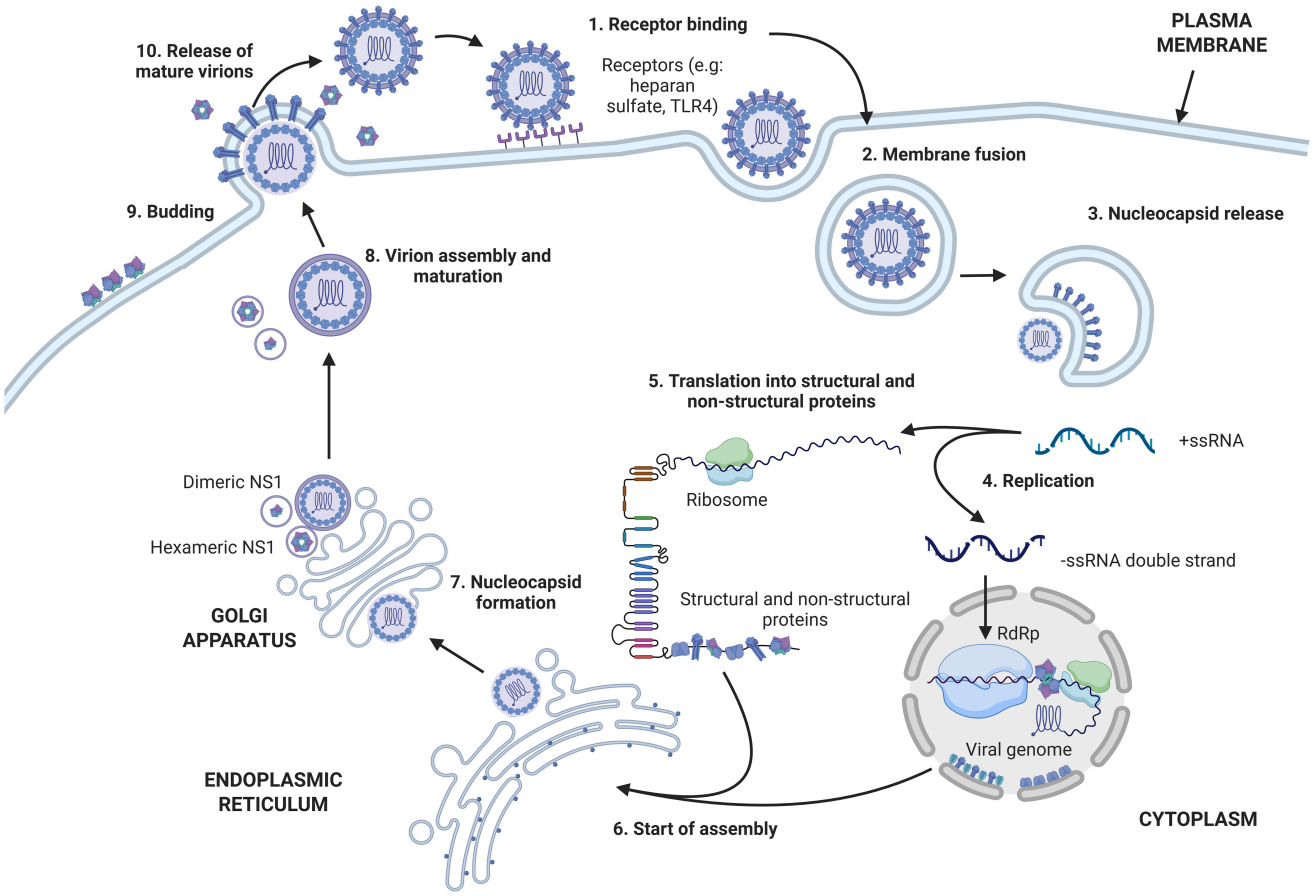
DENV NS1 protein involvement in the life cycle. NS1 plays multiple roles during the DENV life cycle (1–10). After receptor binding and virus entry (1 – 3), (+) ssRNA genome is replicated to produce the (–) ssRNA intermediate that serves as a template for the synthesis of the new (+) ssRNA strand (4). These newly synthesized genomes are also translated into a polyprotein that undergoes proteolytic cleavage to yield both structural and non−structural components (5). Among these, NS1 forms homodimers that associate with the viral RNA replication complex, supporting RNA synthesis and virion assembly through interactions with the prM and E (6 – 8). Due to its hydrophobic and membrane-binding properties, NS1 also contributes to the formation of vesicle packets that house the virus replication machinery. In the secretory pathway, NS1 forms hexamers that are transported through the ER-Golgi network and released as soluble NS1 (9 – 10), distinguishing it from other non-structural proteins. The image was created by Biorender.com .

**Figure 3:**
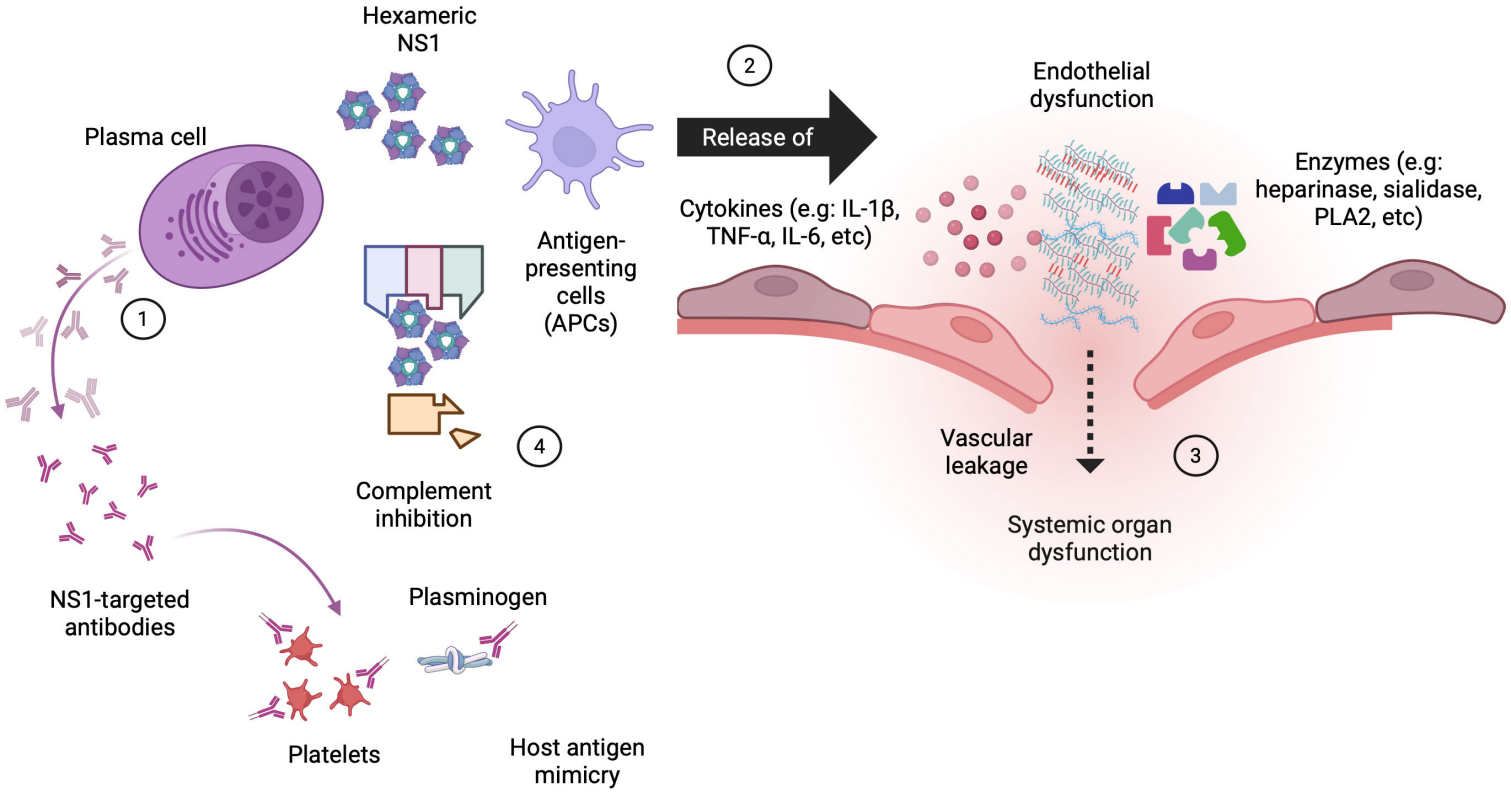
Mechanisms of NS1-mediated immune activation and vascular pathogenesis during DENV infection. Circulating hexameric NS1 drives both immune activation and direct pathogenic effects, contributing to the severe manifestations of dengue. Plasma cells produce NS1-targeted antibodies (1) while antigen-presenting cells (APCs) induce cytokine production (2). The excessive release of pro-inflammatory cytokines such as IL-1β, TNF-α, and IL-6, often accompanied by the release of host enzymes, including heparinase, sialidase, phospholipase A2 (PLA2), collectively causes endothelial dysfunction leading to vascular leakage (3). NS1 also inhibits the complement cascade mechanism by binding to C4, C4b, or C9 (4). NS1-targeted antibodies can cross-react with platelets and plasminogen through molecular mimicry (5). The image was created by Biorender.com.

DENV NS1 is a 48-kDa glycoprotein with a structure that consists of a β-roll, wing domain, and β-ladder domain; each is involved in cell membrane interaction and modulation of host immunity (109, 110). Unlike structural proteins, NS1 is not part of the virion but is secreted as a hexamer and also expressed as a dimer on the surface of infected cells (**Figure 2**) (111–115). DENV NS1 has three forms: intracellular NS1, which supports virus RNA replication; membrane-associated NS1, which aids immune evasion; and secreted NS1 (sNS1), which circulates in the bloodstream. sNS1 plays a prominent pathogenic role by binding to vascular endothelial cells and inducing vascular hyperpermeability through the activation of proinflammatory cytokines and chemokines. NS1 interacts with TLR4 to activate macrophages and endothelial cells, triggering cytokine release and directly disrupting endothelial monolayer integrity (116). sNS1 also activates TLR2 and TLR6 pathways, leading to the upregulation of proinflammatory cytokines such as IL-1β, IL-6, and TNF-α (117). One of the cytokines, macrophage migration inhibitory factor (MIF), appears to contribute significantly to junctional disassembly by promoting the internalization and autophagic degradation of proteins such as VE-cadherin and ZO-1 (118, 119). The internalization or phosphorylation of VE-cadherin and zonula occludens-1 (ZO-1) potentially occurs through clathrin-mediated pathways (120). The degradation and disassembly of the endothelial junctions are further exacerbated by the release of host enzymes, including heparinase, sialidase, and phospholipase A2 (PLA2) (121). This mechanism has been investigated in various endothelial cell models, where exposure to NS1 from all four DENV serotypes led to decreased trans-endothelial electrical resistance (122, 123). In addition, NS1 has been implicated in immune evasion through its interference with the complement system. It can bind directly to complement components C4 and C4b, thereby inhibiting protective complement functions while simultaneously generating excessive activation products such as C3a and C5a (124–126). These complement-activated peptides increase vascular permeability and, together with NS1-mediated disruption of endothelial glycocalyx integrity, drive the vascular leakage characteristic of severe dengue (123, 127) (**Figure 3**).

NS1 has also been implicated in molecular mimicry and autoimmune−related pathogenic effects. The highly conserved C−terminal epitopes share structural similarity with human proteins involved in hemostasis and endothelial integrity, and cross−reactive antibodies can induce endothelial apoptosis, platelet dysfunction, and complement−mediated cytotoxicity (128, 129). Antibodies targeting the C−terminal region of NS1 have been shown to cross−react with host proteins expressed on platelets and endothelial cells, as well as coagulation−associated molecules, potentially contributing to thrombocytopenia, endothelial dysfunction, and vascular leakage in severe dengue (130, 131). Elevated levels of anti−NS1 autoantibodies have been correlated with dengue hemorrhagic fever and dengue shock syndrome, reinforcing the role of NS1 molecular mimicry in disease immunopathogenesis (132, 133).

Recognizing its central role in dengue disease severity, NS1 has been increasingly explored as a candidate antigen for the development of next-generation dengue vaccines (134–138). The optimal structural presentation of the antigen, however, remains a critical consideration. While NS1 naturally transitions from an intracellular homodimer to a secreted hexamer (sNS1) that drives systemic vascular leakage, current strategies prioritize detoxified mutants or truncated fragments to avoid molecular mimicry while maintaining protective epitopes (120, 139, 140). NS1 contains multiple predicted B-cell and T-cell epitopes identified across individual DENV serotypes, supporting its potential as a broadly relevant vaccine target (**Supplementary Figure 1**). Recent work further highlights the potential of displaying conserved T-cell epitopes from the NS1 wing domain to ensure broad HLA coverage across serotypes (120, 139). Human monoclonal antibodies against NS1 have been shown to block endothelial binding and prevent glycocalyx degradation, thereby mitigating vascular leakage (127, 141). These antibodies are expected to functionally block sNS1-mediated TLR4 activation and endothelial damage, necessitating that the vaccine antigen be expressed and subsequently mimic natural sNS1 kinetics during infection (141). In addition, laboratory studies show that Fc-mediated effector functions, including antibody-dependent cellular cytotoxicity (ADCC) and complement-mediated killing of DENV-infected cells, contribute to the reduction of virus and amelioration of NS1-induced pathology (6, 142). Simultaneously, maintaining cell surface expression of the antigen is vital for eliciting these potent ADCC and complement-dependent cytotoxicity (CDC) responses to clear infected cells early in the virus cycle (142, 143). Structural mapping studies further delineated potential pathogenic epitopes on NS1, particularly within the C-terminal region (144–146), while engineered NS1 constructs such as wing-domain (NS1-WD) and truncated or chimeric variants elicit protective immunity without platelet or endothelial binding (122, 137, 147). Mutagenesis strategies further reduce potential cross-reactivity with host proteins while preserving immunogenicity (148). Building on this foundation, together with recent advances in structural mapping and functional profiling assays, a promising framework for a safe NS1-based vaccine design is now emerging. These findings suggest that antigen localization, selection of detoxified NS1 variants, and the induction of balanced humoral and cellular immunity may represent important design considerations for spore-based delivery systems.

Several pre-clinical studies have demonstrated promising results for various NS1-based vaccine platforms in mouse models (**Table 3**). Broad protection was demonstrated using full-length NS1 derived from all four DENV serotypes formulated with the monophosphoryl lipid A (MPLA)/AddaVax adjuvant. Intravenous administration of interferon-α/β receptor-deficient C57BL/6 mice conferred 60 – 100% survival along with significant reductions in both viremia and circulating NS1 antigen levels (134). Moreover, recombinant protein vaccines incorporating NS1 in combination with immune-modulating adjuvants, including the detoxified heat-labile toxin derivative LT_G33D_, have further demonstrated enhanced immunogenicity and protective efficacy (148). Heterologous prime-boost strategies integrating NS1 with live bacterial vectors have also shown enhanced protective immunity. An immunization regimen consisting of NS1 protein priming followed by an oral booster using recombinant *Salmonella* expressing NS1, in the presence of amphotericin B (Amb) as an immune enhancer, significantly augmented both humoral and cellular immune responses. This strategy conferred effective protection against dengue virus challenge in mice, underscoring the potential of mucosal boosting and innate immune modulation to strengthen NS1-directed vaccine efficacy (153). A peptide vaccine targeting a modified NS1 wing domain (NS1-WD), formulated with complete Freund’s adjuvant (CFA), achieved complete survival and significantly reduced viremia, antigenemia, and hemorrhagic manifestations in both C3H/HeN and STAT1^−^/^−^ C57BL/6 mouse models (137). More recently, VLP and nanoparticle-based formulations, such as NS1 1–279 displayed on trimethyl chitosan (TMC) nanoparticles, enhanced humoral immune responses and conferred protection against lethal dengue virus challenge in mice, highlighting the advantages of particulate antigen presentation (149). In parallel, the chimeric construct cEDIII-ΔC NS1 showed improved immune responses, underscoring the potential of antigen design optimization to further strengthen NS1-based vaccine strategies (147).

**Table 3:** DENV NS1-based vaccines at various stages of development.

Platform	Candidate	Phase/stage	References
VLPs	NS11-279TMC NPs	Pre-clinical	(149, 150)
DNA	pcENS1, pcTPANS1	Pre-clinical	(151)
	pD2NS1/pD2NS1+ pIL-2	Pre-clinical	(135)
	pNS (1,3,5)	Pre-clinical	(152)
mRNA	NS1-mRNA	Pre-clinical	(61)
Bacterial vector	*Salmonella*-NS1 (AmB)	Pre-clinical	(153)
Recombinant protein	NS1 and LT_G33D_	Pre-clinical	(148)
	cEDIII-ΔC NS1	Pre-clinical	(147)
	NS1-WD/CFA	Pre-clinical	(137)
	DENV2 NS1 MPLA/AddaVax	Pre-clinical	(134)

The more modern, DNA-based vaccine platforms have also shown favorable outcomes. Dual-plasmid constructs such as pD2NS1/pD2NS1+ pIL-2 conferred 50 – 80% survival in C3H mice, with a 70 – 80% reduction in morbidity, while other DNA vaccines such as pcTPA-NS1 and pcENS1 achieved complete (100%) or substantial protection (86.7%), respectively, in BALB/c mice (135, 136, 138). In addition, multivalent DNA vaccine constructs such as pNS (1,3,5), encoding dengue virus non-structural proteins NS1, NS3, and NS5, have been evaluated at the pre-clinical stage and demonstrated immunogenic potential in experimental models (152). In addition, mRNA-based vaccination encoding NS1 induced robust NS1-specific antibody responses and provided protection in pre-clinical mouse models (61).

## 5 Mucosal vaccines and mucosal immunity

Vaccination remains one of the most effective public health interventions, with thirty-three licensed vaccines currently available for viral and bacterial human diseases (154). Most of these vaccines are delivered via intramuscular or intravenous injection, with only a few formulated for oral administration. Oral vaccines, however, could offer numerous advantages, particularly for pediatric populations, as their ease of administration could significantly improve uptake. Despite these advantages, developing effective oral vaccines remains challenging. Antigens must withstand the stomach’s highly acidic environment, overcome robust mucosal barriers that restrict antigen uptake, and avoid triggering immune tolerance at mucosal surfaces. Immune tolerance, where orally delivered antigens are recognized as harmless, elicits suppressive systemic or mucosal immune responses, an essential mechanism preventing hypersensitivity reactions, that could also diminish the effectiveness of orally administered vaccines (155). Despite these challenges, there are antigens that have been successfully delivered by overcoming the mucosal barrier and circumventing tolerance, particularly when delivered with potent mucosal adjuvants or specialized delivery platforms.

Oral vaccination has been shown to elicit both mucosal and systemic immune responses (156). For example, oral immunization with inactivated *B. subtilis* spores expressing the TonB-dependent receptor (TBDR) antigen of multidrug-resistant *Acinetobacter baumannii* induced antigen-specific immune responses at both mucosal and systemic levels in mice (157). Immunized animals exhibited increased CD4^+^ and CD8^+^ T-cell populations, enhanced B-cell responses, and elevated antigen-specific IgG and IgA in serum and mucosal secretions, which translated into significant protection against bacterial challenge, including marked reductions in pulmonary bacterial burden and preservation of tissue architecture. Complementing these findings, oral administration of *B. subtilis* spores expressing the SARS-CoV-2 spike protein resulted in the induction of antigen-specific systemic and mucosal immune responses with no evidence of adverse effects (158). During natural gut infection, microbial components are recognized by intestinal epithelial cells via pattern-recognition receptors (PRRs) to detect pathogen-associated molecular patterns (PAMPs), leading to the recruitment of antigen APCs such as the intestinal dendritic cells, B cells, and macrophages (159). These APCs then activate downstream mucosal immune cells, initiating innate immune responses and driving adaptive immunity via activation of CD4^+^ and CD8^+^ T lymphocytes. The ability of mucosal immunization to engage APCs and promote systemic lymphocyte activation provides a mechanistic link between mucosal delivery and the induction of protective cellular immunity. Although DENV is not a mucosally transmitted pathogen, this immunological priming is particularly relevant for dengue, where effective protection is increasingly recognized to depend not only on neutralizing antibodies but also on robust T cell-mediated responses. In this context, mucosal vaccine platforms may serve as an upstream strategy to shape the quality and breadth of systemic immunity, including the enhancement of CD8^+^ T cell responses against conserved virus antigens such as NS1.

T cells, particularly CD8^+^ cytotoxic T lymphocytes (CTLs), play a central role in recognizing and eliminating infected cells, thereby limiting virus replication and tissue dissemination (160, 161). Evidence from both human and animal studies indicates that robust dengue-specific T cell responses are associated with lower viremia levels and milder clinical outcomes (160, 162, 163). For instance, individuals mounting cross-reactive CD8^+^ T cell responses against conserved epitopes within non-structural proteins such as NS1, NS3, NS4A/NS4B, and NS5 exhibit enhanced virus clearance and reduced risk of severe dengue (164–167). Therefore, T cell responses offer broader and more durable cross-serotype protection, as they frequently target conserved internal epitopes that are less prone to antigenic variation than those provided by neutralizing antibodies (164). T cell immunity also contributes to immune regulation by modulating cytokine responses and mitigating the immunopathological processes associated with plasma leakage and hemorrhagic manifestations (168). Thus, next-generation dengue vaccines may benefit from a strategy that prioritizes the induction of potent T cell responses to limit virus replication and prevent severe outcomes, rather than solely focusing on preventing virus entry into cells.

Building on the importance of T cell-mediated immunity, NS1 represents a promising T cell antigen. NS1 is abundantly expressed during infection and presented via major histocompatibility complex (MHC) class I pathways, allowing efficient activation of CD8^+^ T cells (169). NS1 also exhibits approximately 70 – 80% sequence conservation across all four DENV serotypes, which strongly suggests its potential for eliciting broad, cross-serotype protection. Incorporating NS1 into vaccine formulations could thus stimulate both humoral and cellular immunity, with the latter playing a decisive role in reducing virus load and disease severity (170). In line with this, NS1-based vaccines have demonstrated substantial protective efficacy in pre-clinical animal models, leading to improved survival outcomes and a significant reduction in clinical disease manifestations (61, 147, 149, 151, 171). These findings emphasize a paradigm shift in dengue vaccine design, moving beyond traditional antibody-focused approaches aimed at preventing infection, toward T cell-oriented strategies that aim to eliminate infected cells and prevent severe disease manifestations. Translating this potential into a viable vaccine will require future research directed at identifying conserved T cell epitopes, optimization of antigen presentation via mucosal vaccine platforms, and development of reliable immunological correlates of T cell-mediated protection.

## 6 Targeting mucosal immunity for dengue vaccination

Although dengue is not traditionally classified as a mucosal pathogen, growing evidence suggests that mucosal surfaces and their associated immune responses play a critical role in both disease pathogenesis and protection (15). Clinical and histopathological studies have demonstrated mucosal involvement during DENV infection, with NS1 binding to the underlying microvascular endothelial cells from the lung and liver vasculature, thereby inducing tissue-specific vascular leakage (111, 172, 173). In addition, gastrointestinal symptoms are frequently observed in severe dengue patients and often correlate with plasma leakage and disease severity, implicating gastrointestinal involvement that may lead to severe disease manifestations (174). Autopsy findings from severe dengue cases often reveal mucosal edema, hemorrhage, and the presence of virus antigen within MALT, including the intestines and nasopharynx (22, 175–179). These findings suggest that mucosal tissues serve not only as sites of virus replication but also as potential targets of DENV NS1-mediated vascular pathologies. Hence, while immune responses elicited at these sites are essential for viral clearance, they may also exacerbate disease severity (116).

The mucosal immune system, particularly in the gastrointestinal and respiratory tracts, comprises organized lymphoid structures such as Peyer’s patches, mesenteric lymph nodes, and nasopharynx-associated lymphoid tissue (NALT). These sites are rich in APCs, including dendritic cells, macrophages, and natural killer (NK) cells, which initiate immune responses by capturing antigens and activating T and B cells (180). A hallmark of mucosal immunity is the production of secretory IgA (sIgA), which can neutralize pathogens extracellularly and intracellularly, preventing virus attachment, entry, and replication at mucosal surfaces (181). IgA antibodies have been detected in dengue-infected individuals and are believed to contribute to early virus neutralization prior to systemic dissemination (182). In contrast, systemic IgG plays a key role in long-term protection and virus clearance (181, 183). In addition to humoral responses, APCs in the gastrointestinal tract capture antigens and present them to major histocompatibility complex (MHC) class I and class II, activating CD8^+^ cytotoxic T cells and CD4^+^ T helper 17 (Th17) cells, respectively (184, 185). These T cell subsets participate in immune surveillance, clearance of infected cells, and cytokine-mediated responses (182, 186).

In light of these observations, the induction of mucosal immunity, particularly through oral or intranasal routes, offers a promising strategy for enhancing protection against DENV by complementing systemic immune responses. While DENV is introduced into the host following a mosquito bite into dermal tissues rather than across respiratory or gastrointestinal mucosa, mucosal vaccination remains immunologically relevant. The rationale does not rest on sIgA intercepting virions at the bite site, but rather on the capacity of mucosal immunization to effectively prime antigen-specific B and T lymphocytes within MALT, which subsequently circulate systemically and contribute to peripheral immune establishment (187). Studies have also demonstrated that mucosal vaccination can establish potent systemic immune memory and generate high titers of circulating IgG and effector T cells capable of responding rapidly to peripheral antigen exposure (188, 189). This systemic arm of mucosal immunization is particularly pertinent for dengue, where pathology occurs in the liver, spleen, and endothelium rather than at mucosal surfaces (190, 191). Recent clinical studies have demonstrated that NS1-specific antibodies, although not neutralizing in the classical pathway, mediate protection by engaging Fc-dependent effector mechanisms such as antibody-dependent cellular cytotoxicity (ADCC). Sanchez-Vargas et al. (142) showed that NS1-directed antibodies can recruit natural killer (NK) cells to lyse infected targets, thereby reducing viral burden and mitigating severe dengue outcomes.

Other pre-clinical studies have also shown promising results, demonstrating that mucosal delivery of DENV antigens could induce both mucosal IgA and systemic IgG responses, along with robust T cell activation (192–194). For example, *Salmonella Typhimurium* lysed through gene E activation was engineered to deliver the envelope protein domain III (EDIII) of all four DENV serotypes using the expression vector pJHL184. Mice immunized with the recombinant *Salmonella* ghosts showed significantly elevated titers of EDIII-specific IgG, IgG1, and IgG2a (p < 0.01), along with increased lymphocyte proliferative activity and CD3^+^ CD4^+^ T-cell subpopulations as compared to non-immunized controls (195). Similarly, a nanoemulsion-based oral vaccine designed to deliver a recombinant tetravalent DENV antigen was able to induce both innate and adaptive immune responses. Immunized mice exhibited robust antigen-specific humoral and cellular responses, characterized by elevated IgG1, IgG2a, and IgA titers, high levels of IFN-γ and IL-4, and increased CD8+ T-cell frequencies (157, 196, 197). In the context of dengue, clinical evidence specific to mucosal vaccines is not yet available, as no licensed oral or intranasal dengue vaccines have advanced into human trials. Nevertheless, established mucosal platforms such as the oral polio vaccine and intranasal influenza vaccine provide proof-of-concept that mucosal routes can induce both mucosal and systemic immune responses with demonstrated clinical efficacy (198, 199). Complementing this, NS1-based dengue vaccine candidates delivered by parenteral routes have shown that circulating antibodies and T cells confer protection against viremia and NS1-mediated vascular leakage in pre-clinical models (116, 134, 140). Thus, mucosal vaccination remains mechanistically relevant by broadening immune activation and reinforcing systemic responses that restrict virus replication, together with effector T-cell functions that kill infected cells, thereby intercepting DENV soon after dermal inoculation. Notably, earlier studies demonstrated oral immunization with food-grade yeast expressing dengue antigens induced both systemic dengue-specific IgG and mucosal secretory IgA (192). In another example, animals orally immunized with a plant-based dengue antigen, tEDIII-Co1, exhibited strong antigen-specific B and T cell responses even in the absence of adjuvants (193).

## 7 Bacterial spores as a mucosal vaccine delivery platform

Gram-positive, spore-forming bacteria, particularly *Bacillus subtilis*, have emerged as robust expression systems and innovative vaccine delivery vehicles (157, 200, 201). These microbes are classified as Generally Recognized As Safe (GRAS) by regulatory agencies, including the Food and Drug Administration (FDA) and European Food Safety Authority (EFSA), due to their non-pathogenic nature and established safety profile in both industrial, food, and probiotic applications (202). Their unique combination of extreme environmental stability, including gastric acidity, while preserving the integrity of surface-displayed antigens, capacity for heterologous antigen presentation, and inherent immunogenicity, positions them as promising candidates for next-generation vaccine platforms (203).

**Figure 4:**
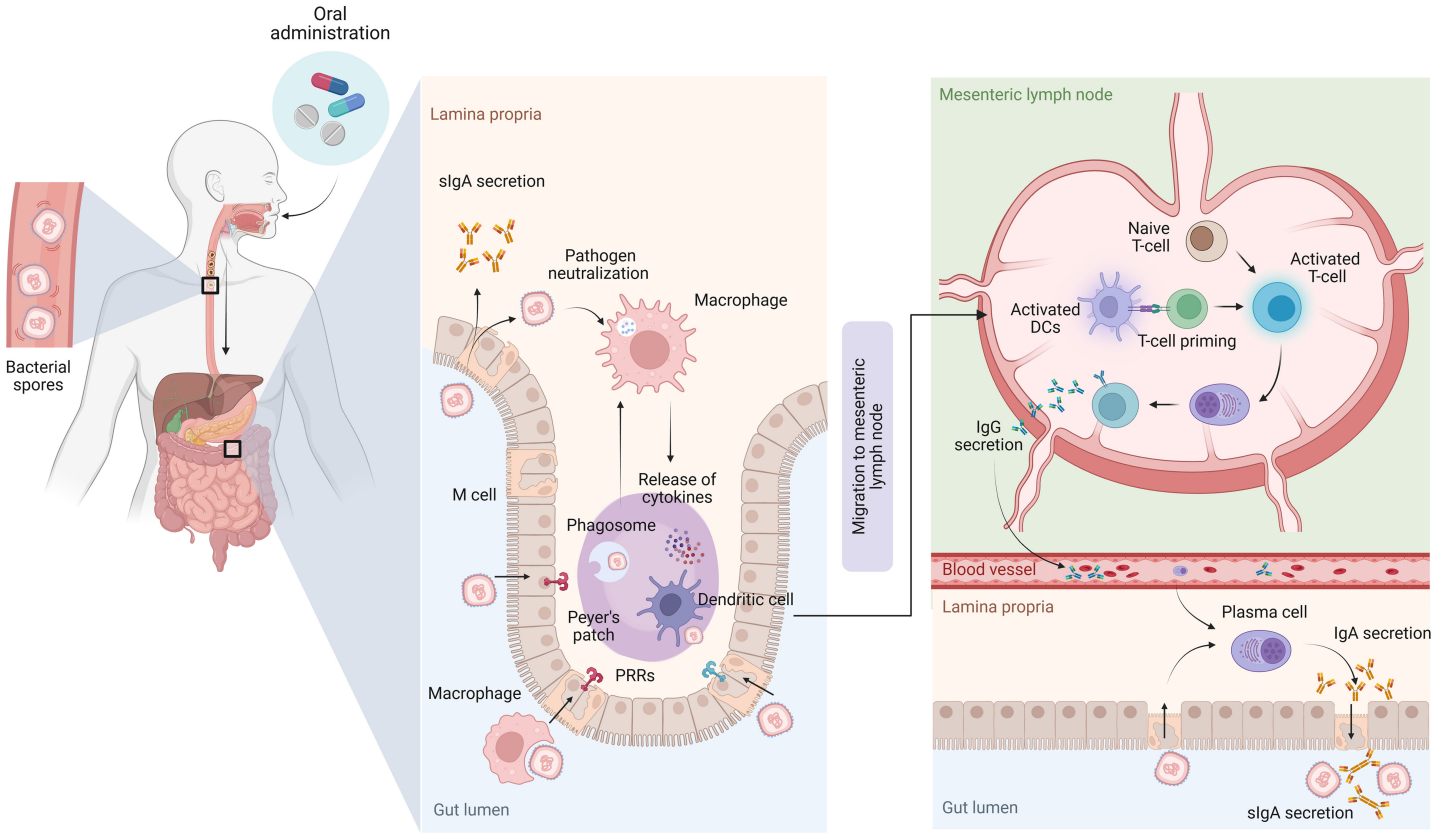
Schematic overview of mucosal and systemic immune activation following oral administration of bacterial spores. Following oral administration, bacterial spores reach the intestinal lumen, where they are taken up predominantly by M cells overlying Peyer’s patches. The spores are subsequently transcytosed into the subepithelial compartment, including the Peyer’s patches and lamina propria, where they are internalized by APCs, such as dendritic cells and macrophages, and processed within phagosomes. Recognition of spore-associated molecular patterns by pattern-recognition receptors (PRRs) triggers the production of pro-inflammatory cytokines and promotes dendritic cell activation and maturation. Activated dendritic cells then migrate from the gut mucosa to the mesenteric lymph nodes, where they present processed antigens to naive T cells, initiating T-cell priming and subsequent B-cell activation, clonal expansion, and antibody class switching. Activated lymphocytes, including effector T cells and antibody-secreting plasma cells, enter the systemic circulation and home to mucosal effector sites. In the lamina propria, plasma cells secrete IgA, which is transported across the intestinal epithelium as secretory IgA (sIgA). The released sIgA accumulates in the intestinal lumen, where it contributes to pathogen binding and neutralization. This schematic depicts a proposed and representative pathway of spore-mediated immune activation and does not encompass all mechanisms involved in gut antigen sampling or immune regulation. The image was created by Biorender.com.

The resilience of bacterial spores stems from their complex, multi-layered structure formed during the sporulation process, which is initiated under nutrient-depleted growth conditions. This transformation results in metabolically dormant spores capable of withstanding extreme conditions, including moist heat (121 °C for 15 minutes), desiccation, ultraviolet (UV) radiation, and chemical disinfectants (204). Its architecture comprises a core containing DNA stabilized by small acid-soluble spore proteins (SASPs) and dipicolinic acid (DPA), an inner membrane with low permeability, a thick cortex composed of cross-linked peptidoglycan, and a proteinaceous outer shell consisting of over 70 distinct proteins (204, 205). These biophysical properties confer several advantages for vaccine applications. The stability of spores, including resistance to gastric acidity (pH 1.5-3), bile salts (0.3-2%), and prolonged desiccation, facilitates potential oral delivery by eliminating the necessity for cold chain infrastructure, thereby addressing a major logistic barrier in global vaccine distribution in the low- and middle-income countries (LMICs). Upon ingestion, spores are taken up by M cells in Peyer’s patches within the gut-associated lymphoid tissue (GALT), facilitating antigen uptake and immune system priming (**Figure 4**) (206). Spore germination within the intestinal lumen promotes the gradual release of antigen, thereby prolonging immune system exposure and potentially reducing the need for multiple booster doses (207). While mucosal interaction triggers the production of secretory IgA, a key biomarker for successful immunization, it also simultaneously engages systemic immunity. By activating T and B lymphocytes, this process generates circulating IgG antibodies and establishes long-term immunological memory (208). This pharmacokinetic profile differs from that of parenteral vaccines, where antigens are delivered directly into systemic tissues and efficiently captured by local antigen-presenting cells (APCs). These APCs process and present antigen to MHC class I and II complexes, leading to activation of humoral and cellular immune responses. This results in immunity that is largely systemic, with efficient production of effector and memory lymphocytes that provide protection against disseminated infection. Parenteral vaccination, therefore, primarily drives rapid systemic immune induction but lacks sustained antigen exposure across multiple immune compartments.

Pre-clinical studies of spore-based vaccines targeting non-dengue pathogens demonstrated the effective induction of antigen-specific CD4^+^ and CD8^+^ T-cell responses alongside mucosal and systemic immune responses following oral administration. This dual stimulation of mucosal and systemic immunity highlights the versatility of the bacterial spore-based platforms, although systemic responses remain particularly critical for dengue. (136). In addition, bacterial spores also exhibit intrinsic adjuvant properties, by which components of the spore coat and germination-derived molecules stimulate pattern recognition receptors, including Toll-like receptors (TLRs) 2 and 4, triggering dendritic cell maturation and macrophage activation. These innate immune responses favor the development of Th1/Th17-polarized adaptive responses, which are particularly effective against intracellular pathogens (209–212).

Bacterial spore surfaces can be engineered for antigen presentation through genetic fusion to the spore coat proteins, including but not limited to CotB, CotC, CotG, and CotZ. This is achieved through recombinant expression in the mother cell during sporulation, chromosomal integration, or passive adsorption of spores with stably surface-bound antigen based on hydrophobic or electrostatic interactions (213–216). Building on these approaches, subsequent studies have shown that antigen localization to the spore surface can be achieved independent of Cot protein fusion, thereby simplifying strain construction and expanding the versatility of spore-based vaccine platforms (157, 158, 196). Pre-clinical studies have demonstrated the efficacy of spore-based vaccines across multiple pathogen targets. Recombinant spores expressing the protective antigen of *B. anthracis* induced neutralizing antibody levels comparable to conventional anthrax vaccines (217). More recently, *B. subtilis* spores engineered to express SARS-CoV-2 spike protein (158) and receptor-binding domain (RBD) (201) were demonstrated to elicit adaptive immune responses and significantly increased neutralizing antibodies in mice and human volunteers. *B. subtilis* spores displaying *Acinetobacter baumannii* TonB-dependent receptors (TBDRs) elicited robust antigen-specific immune responses in both intestinal mucosa and sera following oral administration (157, 196). These bacterial spore-based vaccines are characterized by high thermostability, as lyophilized spores preserve their viability and immunogenic potential for up to 12 months at 25 °C (218).

Although *B. subtilis* spores have been widely explored as mucosal delivery vehicles for various antigens, there is currently no dengue-specific functional data demonstrating NS1-spore constructs in pre-clinical models. Future studies to validate spore-based NS1 constructs in dengue animal models, particularly in terms of T-cell functionality, tissue-specific viral control, and comparative performance against existing NS1 platforms, should be emphasized. However, proof-of-concept evidence supports the feasibility of oral NS1 vaccination. Liu et al. (153) reported that a regimen consisting of parenteral NS1 immunization followed by an oral *Salmonella* expressing NS1 booster, administered with amphotericin B as an immune enhancer, significantly elevated both humoral and cellular immunity. This finding highlights the potential of gut-primed enhancement and innate immune activation to improve NS1-targeted vaccine immunogenicity and confer protection against DENV (153). Oral administration of recombinant *B. subtilis* spores expressing NS1 could stimulate mucosal dendritic cells and M cells, leading to the priming of mucosal-homing T cells and subsequent activation of systemic CTL responses (219). While DENV initially replicates in skin-resident immune cells, mucosal vaccination offers a strategic advantage by broadening systemic immunity, thereby limiting virus dissemination. The ability to induce both mucosal and systemic T cell immunity positions spore-based vaccines as a powerful strategy not only for infection control but also for mitigating disease severity. GRAS-certified *B. subtilis* spores, hence, represent a convergence of safety, biological durability, immunological versatility, and delivery flexibility. Their capacity to elicit both mucosal and systemic immunity, coupled with thermal stability and flexible antigen presentation strategies, makes them a promising platform for vaccine development. In addition, mucosal vaccines could also provide several advantages over parenteral vaccines in dengue prevention, including needle-free, easier administration, and convenience for repeated dosing, which may improve patient willingness to receive the vaccine, especially in dengue endemic regions (180).

## 8 Future perspectives and challenges

Despite promising advances in spore-based dengue vaccine development, particularly those targeting NS1, several challenges remain for its clinical translation. DENV NS1 contributes to severe dengue pathology and vascular leakage through several mechanisms, including complement activation and endothelial disruption. Hence, NS1 itself may pose a potential threat of exacerbating the severity of dengue. In addition to the strategy of truncating NS1 by removing the offending carboxyl end of NS1, the method to deliver the NS1 expressed on the spore surface would mitigate the potential pathologic effects, as the protein would not interact directly with the endothelium nor activate the complement pathways. NS1-based candidate oral vaccines, however, have been reported to elicit antibodies that mediate Fc-dependent effector functions, such as ADCC, and also induce functional cell-mediated immunity that will facilitate the killing of infected cells. Collectively, these findings support the utilization of NS1 into spore-based mucosal platforms as a means to harness protective immunity while mitigating NS1-associated pathologies. A major hurdle, however, is the optimization of antigen expression on or within the bacterial spore coat to ensure consistent immunogenicity without compromising spore stability. While *Bacillus subtilis* spores are inherently safe and robust, scalable production under good manufacturing practice (GMP) conditions remains underexplored (158, 220). This limitation is primarily due to the need for industrial-scale sporulation, which often results in heterogeneous spore populations, variable recombinant yield, and potential inconsistent vaccine potency between batches. Consequently, downstream processing, including purification and validation of antigen expression, becomes essential to standardize the final preparation. These processes, however, introduce additional complexities relative to conventional subunit or inactivated vaccine platforms. On the other hand, while parenteral vaccines require extremely pure antigens, oral spore-based vaccines could remain immunologically active without requiring near-complete recombinant spores, potentially requiring a simpler manufacturing system, as they require no additional purification steps. This is because effective mucosal and systemic immune responses have been observed even when antigen expression is confined to a 20 – 30% subset of recombinant spores (157, 196).

Apart from that, the lack of standardized quality control assays for spore-based vaccines further complicates their translation to clinical use. The development of assays to validate recombinant spore proportion and spore viability would therefore be invaluable in ensuring product consistency and regulatory compliance. While manufacturing consistency and quality control remain significant hurdles, the lack of a clear regulatory framework for spore-based vaccines presents an equally critical barrier to clinical translation. This ambiguity stems from their hybrid biological nature. Recombinant *B. subtilis* spores engineered to express antigens do not align with existing categories of food, probiotic, or biological vaccines. Although *B. subtilis* is classified as “Generally Recognized as Safe” (GRAS) by the U.S. Food and Drug Administration for consumption, its recombinant derivatives constitute genetically modified biological products with immunological functions, thereby extending beyond the scope of food-grade regulation. Classifying such formulations strictly as vaccines, however, overlooks their oral delivery and food-compatible characteristics. This regulatory gap highlights the potential need for an intermediate classification, perhaps termed “food-grade vaccine” or “oral biotherapeutic”, which reflects their dual identity as safe ingestible vectors with defined immunological function. Formalizing such a category would facilitate more tailored safety assessments and standardized evaluation criteria, ultimately streamlining the clinical translation of spore-based technologies.

Beyond regulatory constraints, the complexity of mucosal immune responses poses an additional challenge to the development of spore-based dengue vaccines. While mucosal vaccination has the potential to elicit both humoral and cell-mediated immune responses, the magnitude and quality of the responses could vary substantially across individuals and populations. Such variabilities may be influenced by host-associated factors such as pre-existing microbiota composition, nutritional status, and genetic background. Moreover, there are raised concerns regarding the potential induction of immune tolerance following mucosal immunization, particularly given the commensal nature of *B. subtilis* within the gastrointestinal tract. Nevertheless, the inherent adjuvant properties of *B. subtilis* spores offer a promising means to overcome this limitation. The spores are capable of engaging TLRs and promoting TLR-mediated cytokine secretion, favoring immune activation over tolerance. Further optimization of antigen display density, dosing intervals, and spore formulation could enhance mucosal immunogenicity while minimizing the risk of tolerance induction.

Since *B. subtilis* spores are recognized as GRAS and have a long history of use in foods and probiotics, their use as a vaccine delivery platform is considered safe. A recent study demonstrated that *B. subtilis* spores, including inactivated preparations, showed no signs of toxicity following both *in vitro* and *in vivo* administrations, even at the highest dose of 1 x 10^10^ CFU/mL (158). Hence, the safety of spore-based formulations supports the possibility of repeated oral administration, allowing flexible boosting strategies to sustain or enhance immune responses over time without the safety concerns typically associated with repeated dosing of conventional vaccines.

Establishing an effective dosing strategy for orally delivered spore-based dengue vaccines would be another challenge. While high-dose formulations, such as those containing approximately 100 billion spores per dose, have demonstrated enhanced immunogenicity, excessive antigen exposure may inadvertently induce mucosal tolerance, whereas lower doses may elicit suboptimal protection. Dose-response studies are therefore essential to define the immunological threshold that maximizes efficacy without compromising immune balance. In parallel with dose optimization, formulation strategies should also consider the integration of multiple dengue antigens or serotypes to achieve broader and more durable protection. In this context, the use of NS1 offers a promising approach, as it would circumvent the issue of ADE that has for decades plagued the development of dengue vaccines. Nonetheless, further studies are warranted to elucidate any potential detrimental role of NS1-antibody complexes in severe dengue.

In summary, a mucosal dengue vaccine delivered utilizing a GRAS bacterial spore-based platform represents a promising strategy to improve dengue prevention by inducing safe and balanced immune responses while minimizing risks associated with ADE. Spore-based NS1 vaccine could be particularly valuable in dengue-endemic regions, as it has the potential to overcome key limitations of current dengue vaccines and reduce the burden of severe dengue in endemic populations, especially those in low- and middle-income countries, where affordable, ease and needle-free delivery, low-cost and scalable production, and easily deployable dengue vaccines are urgently needed.

## Supplementary material

Supplementary Figure 1 Predicted T-cell and B-cell binding epitopes of DENV NS1 across all DENV serotypes. Epitope mapping was performed using NS1 sequences from DENV-1 (NP_722461.1), DENV-2 (NP_739584.2), DENV-3 (YP_001531169.2), and DENV-4 (NP_740318.1). Predicted T-cell epitopes are shown in the upper panel, while predicted B-cell epitopes are shown in the lower panel. Epitope predictions were generated using IEDB analysis tools.
