# Supplementary figures and images for: Targeting dengue through mucosal vaccination: the potential of NS1 and bacterial spore-based delivery platforms
Source: Front Immunol. 2026 Jul 14;17:1809605. doi: 10.3389/fimmu.2026.1809605 (PMC13407787; doi:10.3389/fimmu.2026.1809605)

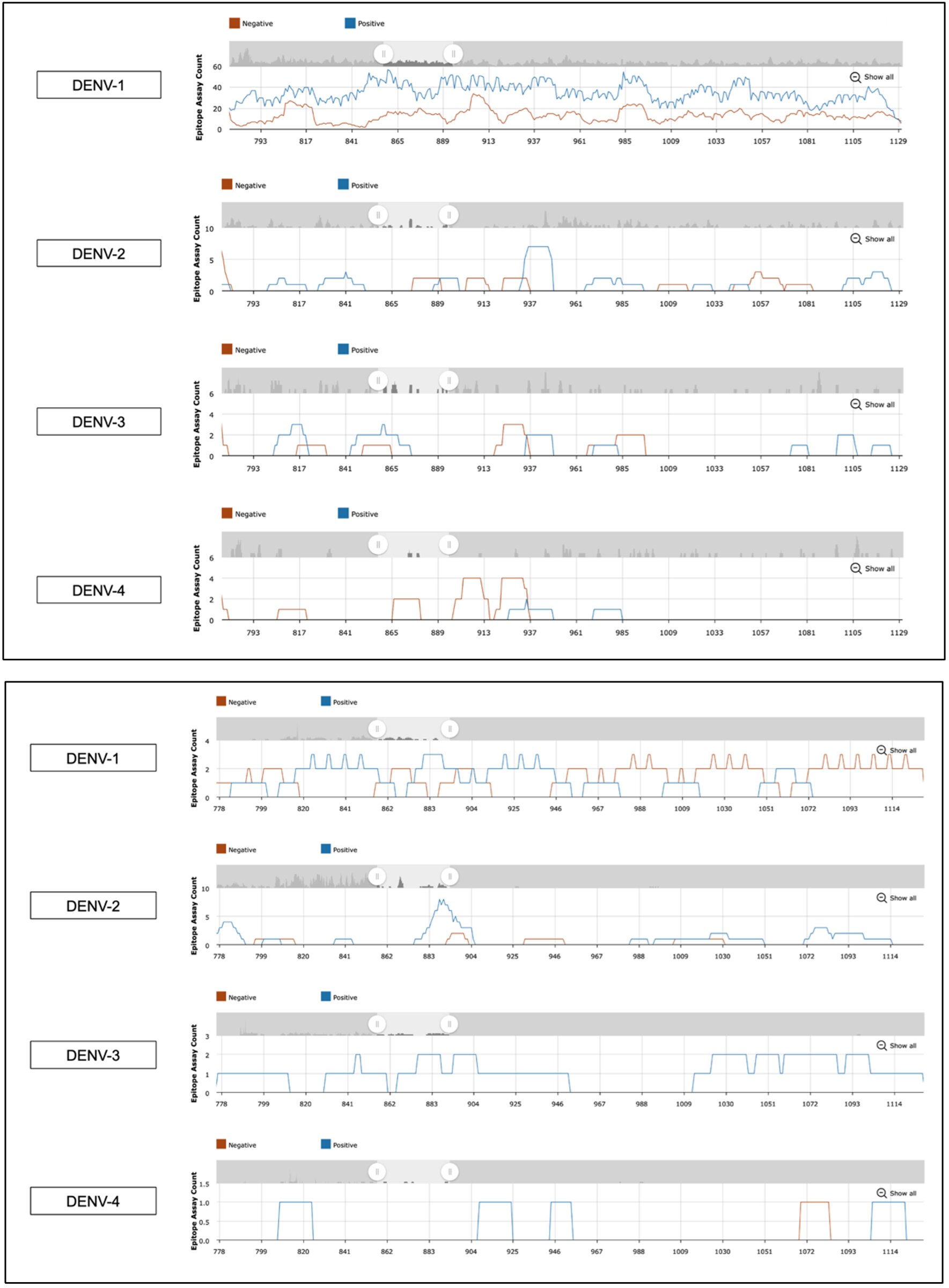

Supplement: Supplementary Figure 1 — Predicted T-cell and B-cell binding epitopes of DENV NS1 across all DENV serotypes. Epitope mapping was performed using NS1 sequences from DENV-1 (NP_722461.1), DENV-2 (NP_739584.2), DENV-3 (YP_001531169.2), and DENV-4 (NP_740318.1). Predicted T-cell epitopes are shown in the upper panel, while predicted B-cell epitopes are shown in the lower panel. Epitope predictions were generated using IEDB analysis tools. [file Image1.jpg]
